# Cycling among people experiencing socio-economic disadvantage: a scoping review protocol

**DOI:** 10.12688/hrbopenres.14005.1

**Published:** 2025-01-23

**Authors:** Louise Foley, Shauna O'Mahony, Yvonne Ryan-Fogarty, Catherine B Woods, Katie Robinson, Colin Fitzpatrick, James Green

**Affiliations:** 1 School of Allied Health, University of Limerick, Limerick, V94 T9PX, Ireland; 2 Physical Activity for Health Research Centre, Health Research Institute, University of Limerick, Limerick, V94 T9PX, Ireland; 3 Department of Chemical Sciences, University of Limerick, Limerick, V94 T9PX, Ireland; 4 Department of Physical Education & Sport Sciences, University of Limerick, Limerick, V94 T9PX, Ireland; 5 Ageing Research Centre, Health Research Institute, University of Limerick, Limerick, V94 T9PX, Ireland; 6 Department of Electronic & Computer Engineering, University of Limerick, Limerick, V94 T9PX, Ireland

**Keywords:** active travel; social inclusion; micromobility; low SES; health inequity; transport

## Abstract

**Introduction:**

Active mobility, such as walking, wheeling, and cycling, is a low-carbon transport mode and a source of physical activity. Cycling, as a form of active mobility, is associated with physical and mental health benefits, transport cost savings, and improved air quality. During the transition to sustainable mobility, equitable outcomes depend on opportunities for active mobility reaching across our societies. This review will chart what is currently known about cycling among people experiencing socioeconomic disadvantage.

**Objective:**

To conduct a scoping review to understand the extent and type of evidence reporting utility cycling (i.e., cycling for transport) among people experiencing socioeconomic disadvantage.

**Inclusion criteria:**

This review will include studies involving adult participants who are described as experiencing socioeconomic disadvantage. Studies will be included if the focus/objective relates to utility cycling and the study reports cycling experiences, purposes, barriers, enablers, frequencies, perceptions, correlates, determinants, impacts, and/or interventions. Primary research using a qualitative, quantitative, or mixed-method design will be considered. Relevant peer-reviewed articles, conference proceedings, dissertations/theses, and preprints will be included.

**Methods:**

The review will be conducted in accordance with the Joanna Briggs Institute’s guidance for scoping reviews. A search strategy that includes key terms and subject headings was developed and translated for use across the following databases: PsycINFO (via EBSCOhost), SPORTDiscus (via EBSCOhost), CINAHL (via EBSCOhost), Embase, PubMed, Scopus, ProQuest Dissertations and Theses (via Web of Science), Europe PMC (preprints), and OpenAlex (preprints). Database search results will be collated in Zotero and uploaded to Covidence for study selection. Titles, abstracts, and subsequent full texts will be independently screened by two reviewers. Data will be extracted from the included studies using a data-extraction tool. Data will be synthesized in two stages: (1) charting the data and (2) descriptive qualitative content analysis.

## Introduction

How people move between places has consequences for individual health, population health, and environmental health. Car use is associated with carbon emissions, air pollution, sedentarism, resource extraction, and land use demand ( Miner *et al.*, 2024). Nevertheless, this mode of transport predominates, with an increase of 6.7% in passenger cars registered in the European Union between 2018 and 2023 ( Eurostat, 2023). In Ireland, almost 70% of trips are made by car, compared to 19% for walking and 2% for cycling, with almost half (49%) of the total trips by all modes covering less than 5 km ( National Transport Authority, 2023). The scope to replace car use with more sustainable and healthy modes is therefore apparent, while the need to do so for human and environmental health is imperative.

Active travel modes can contribute to reducing some of the negative impacts of car use. From a health perspective, active commuting is associated with reduced risk of mortality, cardiovascular disease, and diabetes ( Dinu *et al.*, 2019). A 2011 review concluded that attempts to promote bicycle use contributed to improving population health ( Oja *et al.*, 2011), while a later meta-analysis reported a 10% reduction in the risk of all-cause mortality when cycling reached recommended physical activity levels ( Kelly *et al.*, 2014). The same review suggested that the greatest benefits could be observed by promoting cycling among people with low activity levels ( Kelly *et al.*, 2014). Beyond physical health, biking has also been reported to be associated with mental, social, and financial benefits ( Green *et al.*, 2021).

Cycling is a low-carbon travel mode, with cyclists’ total daily travel producing 84% fewer CO _2_ emissions than non-cyclists ( Brand *et al.*, 2021). Even in the context of increasing electric vehicle (EV) ownership, cycling remains an important component of decarbonizing the transport sector. For example, an equity gap has been identified in EV adoption in Ireland, with less affluent and lower-income areas installing fewer household chargers ( Caulfield *et al.*, 2022). Alongside the social injustices associated with inequitable adoption, replacing internal combustion engine vehicles with EVs upholds other issues with dominant private vehicle use, such as traffic congestion ( Marsden, 2023). Therefore, promotion of affordable, low-carbon, active mobility is necessary for an inclusive energy transition in the transport sector.

Equity has been identified as a factor influencing the relationship between transport and health outcomes ( Glazener *et al.*, 2021). In a non-systematic review of inequalities in bicycle use, it was reported that cycles were used more by people with higher incomes and higher levels of education completion, although variations across contexts were found ( Jahanshahi *et al.*, 2020). In Germany, a large-scale study on short trips reported that cycling was significantly associated with higher education levels ( Hudde, 2022); whereas in a UK study, low-cycling areas showed a trend towards higher rates of cycling among people with lower levels of education completion ( Lawlor *et al.*, 2021). In a study conducted in Canada, the modal share for cycling to work was reported to be significantly higher in high-income areas ( Fuller & Winters, 2017). Therefore, the association between cycling and socioeconomic disadvantage is complex. Indeed, the extent to which participation in active mobility is influenced by choice or necessity has been considered ( Salvo *et al.*, 2023).

Previous studies have explored issues related to cycling, specifically in low-income settings. In a lower-income minority neighborhood in the US, wider cycle tracks and secure home bike parking were identified as investment targets ( Lusk *et al.*, 2017). In a low-income suburban area in Canada, lower-income households more commonly reported expense and security concerns as factors influencing cycling participation, while higher-income households were more likely to report health and safety as relevant ( Ledsham *et al.*, 2023). In a study of psychological factors associated with cycling in disadvantaged neighborhoods in China, social norms were related to cycling behavior ( Ma *et al.*, 2021). Initiatives to promote active mobility should, therefore, be designed to meet contextually relevant needs and tailored to prevent exacerbation of existing inequalities ( Lawlor *et al.*, 2021).

Hansmann *et al.* (2022) recommend accounting for equity in the development and evaluation of active travel interventions. Charting the available evidence on cycling among people experiencing socioeconomic disadvantage is therefore necessary to guide future research and practice in this area. This protocol describes a scoping review of the existing literature on this topic. A preliminary search of titles on Scopus, the Cochrane Database of Systematic Reviews, and *JBI Evidence Synthesis* was conducted on 20 ^th^ June 2024, and no existing or ongoing systematic or scoping review on the topic was identified. Scoping the existing evidence on this topic can inform future systematic reviews of the literature and chart the types of experiences, correlates, and interventions examined to date.

## Review questions

### Primary review question

•    What is the extent and type of evidence available regarding utility cycling among people experiencing socioeconomic disadvantage?

### Secondary review questions

•    What types of factors associated with utility cycling have been studied among people experiencing socioeconomic disadvantage?

•    What types of interventions have been implemented to promote utility cycling among people experiencing socioeconomic disadvantage?

•    What types of outcomes of cycling have been examined among people experiencing socioeconomic disadvantage?

•    What are the gaps in current evidence and priorities for future research on cycling among people experiencing socioeconomic disadvantage?

## Inclusion criteria

### Participants

Studies involving adult participants aged 18 years or older with a population defined by the original study authors as experiencing socioeconomic disadvantage (e.g., low income, low socioeconomic status, experiencing poverty) will be included. Studies involving children will be excluded unless the focus is explicitly on parents’ transportation of children.

### Concept

Studies will be included if the study objective and/or focus explicitly relate to utility cycling, that is, cycling for transport, and reports one or more of the following aspects: experiences, purposes, barriers, enablers, frequencies, perceptions, correlates, determinants, impacts, and/or interventions. Studies will be excluded if cycling is only reported for a different purpose in the research, for example, physiological testing or warm-up exercise.

### Context

Studies conducted in low-, middle-, and high-income countries will be considered for inclusion.

### Types of sources

Primary research using a qualitative, quantitative, or mixed-method design will be included. Peer-reviewed articles, preprints, theses, and conference papers will be considered. Commentaries, opinion pieces, editorials, and letters will be excluded from the review. Studies reported in languages other than English will be excluded.

## Methods

The scoping review will be conducted in accordance with the Joanna Briggs Institute (JBI) methodology for scoping reviews ( Peters *et al.*, 2020a; Peters *et al.*, 2020b) and reported according to the PRISMA extension for scoping reviews (PRISMA-ScR; Tricco *et al.*, 2018). This protocol is reported according to the JBI template for scoping review protocols ( Peters *et al.*, 2020a). The nine stages for scoping reviews outlined by Peters *et al.* (2020a) will be followed (see Table 1). The review was prospectively registered on the Open Science Framework on 26 ^th^ July 2024: https://doi.org/10.17605/OSF.IO/26KYD

**Table 1.** Scoping review stages (from Peters *et al.*, 2020a).

#	Stage
1	Defining and aligning the objective/s and question/s
2	Developing and aligning the inclusion criteria with the objective/s and question/s
3	Describing the planned approach to evidence searching, selection, extraction, presentation
4	Searching for the evidence
5	Selecting the evidence
6	Extracting the evidence
7	Analysis of the evidence
8	Presentation of the results
9	Summarizing the evidence in relation to the purpose of the review, making conclusions and noting any implications of the findings

### Search strategy

The search strategy was designed to locate peer-reviewed articles and selected grey literature. A three-step process was used to develop a search strategy for this review. First, an initial limited search in PsychINFO (via EBSCOhost) and SPORTDiscus (via EBSCOhost) was conducted to identify articles on the review topic. Keywords contained in the titles and abstracts of relevant articles and the index terms attached to these articles were identified and used to develop a full search strategy that was peer reviewed by a librarian. The search terms were then translated for use across the following databases: PsycINFO (via EBSCOhost), SPORTDiscus (via EBSCOhost), CINAHL (via EBSCOhost), Embase, PubMed, Scopus, ProQuest Dissertations and Theses (via Web of Science), Europe PMC, and OpenAlex. Before it was finalized, the search strategy was validated using a set of key papers that were prospectively identified as relevant for inclusion. An example of the search strategy for Embase is presented in Table 2. If feasible, forward and backward citation searches will be conducted for the included studies to identify any additional records that were not identified in the database search.

**Table 2.** Sample search strategy.

**Database**	Embase
**Limiters**	None
**Date of sample search**	10/07/24
**Search terms including key words** ** and index terms combined with** ** Boolean operators**	( (bicycl* OR cyclist* OR cycling OR cycled OR bike* OR biking OR e- bik* OR ebik* OR pedelec OR bikeshare):ti,ab OR ‘cycling’/exp/mj ) AND ( (disadvantage* OR socioeconomic* OR poverty OR “low* income” OR “low* SES” OR “low* educat*” OR deprived OR underserved ): ti,ab OR ‘social class’/exp/mj OR ‘socioeconomics’/exp/mj )
**Number of records retrieved**	1614

The databases to be searched for peer-reviewed articles include PsychINFO (EBSCOhost), CINAHL (EBSCOhost), SPORTDiscus (EBSCOhost), Embase, Scopus, and PubMed. The grey literature that will be considered includes preprints, conference papers, and theses/dissertations. Preprints will be searched in Europe PMC and OpenAlex using the preprint filter, conference papers will be searched in Scopus as part of the article search, and theses and dissertations will be searched in Web of Science by selecting the ProQuest Dissertations and Theses Citation Index. As the use of translation services is not feasible in the current project, only studies published in English will be included in the review. Databases will be searched from their inception to the date of the final search.

### Study selection

Following the search in each database, all identified records will be exported to Zotero (version 6) for collation and then imported into Covidence (2024 version) review software. Duplicate records will be removed using the Covidence de-duplicate function. Following a pilot screen of the first 5% of the records listed by author, titles and abstracts will be screened by two independent reviewers (LF, SO’M). Potentially relevant studies will be retrieved in full and imported into the Covidence database. Full texts will be assessed in detail by two independent reviewers (LF, SO’M) using the inclusion criteria. Any conflicts at each stage of the study selection process will be resolved through discussion or with consensus from another reviewer (JG). The results of the search and study selection process, including reasons for exclusion of full texts, will be reported in the final scoping review and presented in a PRISMA flow diagram ( Page *et al.*, 2021).

### Data extraction

Data will be extracted by one reviewer (LF or SO’M) and cross-checked for accuracy by a second reviewer (SO’M or LF). Any conflicts between the reviewers will be resolved through discussion or with consensus from another reviewer (JG). The extracted data will include details about the participants, concepts, contexts, study methods, and key findings relevant to the review questions. The data extraction procedure will be piloted for at least two of the included studies. The draft data extraction tool (see Extended Data: https://doi.org/10.17605/OSF.IO/SZD42) may be modified and revised as necessary during the process of extracting data from each study. All modifications and revisions will be detailed in the final scoping review. The extracted data items for each included study will be openly shared on the OSF. 

### Data analysis

Data will be synthesized in two stages to understand the extent and type of evidence reporting utility cycling among people experiencing socioeconomic disadvantage:

1. Charting the data: Frequency counts and descriptive characteristics, such as year, country, setting, and participant characteristics, will be presented in a tabular format.
2. Descriptive qualitative content analysis: Inductive content analysis will be conducted following the guidance of Pollock *et al.* (2023) to synthesize data across multiple study designs in scoping reviews. The following stages will be completed: immersion in the data, inductive extraction and analysis, open coding, development of the coding framework, extraction and organizing, and categorization. The results of the content analysis will be presented narratively, including a descriptive summary of the focus and the findings of the included studies.

## Conclusions

This scoping review aims to chart what is known about utility cycling among people experiencing socioeconomic disadvantage. The results will highlight the form and extent of literature on the topic, with a focus on the types of factors associated with cycling that have been explored, the types of interventions that have been delivered, and the types of outcomes that have been assessed. Findings from this review should highlight gaps in current evidence and may identify avenues for future research, such as systematic reviews, on this topic. The review is being conducted as part of a larger project that aims to design and evaluate a cycling intervention for communities experiencing socioeconomic disadvantage in Ireland.

## Ethics and consent

Ethics and consent were not required.

## Data Availability

The list of records obtained from the database searches and extracted items for each included study will be shared openly on Open Science Framework. The results of the synthesis will be described in the final scoping review. Open Science Framework: Cycling among people experiencing socio-economic disadvantage: a scoping review. https://doi.org/10.17605/OSF.IO/SZD42 ( Foley & Green, 2024) The project contains the following extended data: Protocol supplementary file 1 _ Data extraction tool v1.docx Data are available under the terms of the Creative Commons Attribution 4.0 International license (CC-BY 4.0) ( https://creativecommons.org/licenses/by/4.0/) This scoping review protocol is reported according to recommended items to address in a scoping review protocol ( Peters *et al.*, 2022). A completed checklist is available on: Open Science Framework: Cycling among people experiencing socio-economic disadvantage: a scoping review. https://doi.org/10.17605/OSF.IO/SZD42 ( Foley & Green, 2024) The project contains the following reporting checklist: Protocol supplementary file 2 _ Recommended items to address in a scoping review protocol - checklist .docx Data are available under the terms of the Creative Commons Attribution 4.0 International license (CC-BY 4.0) ( https://creativecommons.org/licenses/by/4.0/)
