# Supplementary material for: Cycling among people experiencing socio-economic disadvantage: a scoping review protocol
Source: HRB Open Res. 2025 Jan 23;8:7. [Version 1] doi: 10.12688/hrbopenres.14005.1 (PMC11876797; doi:10.12688/hrbopenres.14005.1)
Supplement: Peer review history

# Reviewer report: Reviewer response for version 1

**DOI:** 10.21956/hrbopenres.15371.r45725

**Published:** 2025-03-03

**Authors:** Ashish Joshi

**Affiliations:** 1 University of New Mexico, Albuquerque, USA

The description is crisp and easy to follow. 

 I have a few comments as follows:

 - while the primary review question examines the extent of utility cycling, it might be worth examining the availability/access to biking infrastructure as a factor among people experiencing socioeconomic disadvantage.

 - additionally, for descriptive qualitative content analysis, what tools are the authors considering?

 - the authors mention considering commentaries, opinion pieces, editorials and letters along with peer reviewed articles particularly since opinion pieces and editorials may introduce different biases compared to peer-reviewed research. do the authors intend to analyze subjective pieces separately from the peer reviewed articles? if so, it might be worth mentioning how. and if not, then why not?

Is the study design appropriate for the research question?

Yes

Is the rationale for, and objectives of, the study clearly described?

Yes

Are sufficient details of the methods provided to allow replication by others?

Yes

Are the datasets clearly presented in a useable and accessible format?

Not applicable

Reviewer Expertise:

Bicycle safety, transportation planning, transportation engineering, active transportation

I confirm that I have read this submission and believe that I have an appropriate level of expertise to confirm that it is of an acceptable scientific standard.

# Reviewer report: Reviewer response for version 1

**DOI:** 10.21956/hrbopenres.15371.r45204

**Published:** 2025-02-17

**Authors:** Angela Curl

**Affiliations:** 1 University of Otago, Dunedin, New Zealand

The article outline a protocol for a scoping review focussed on cycling among people experiencing socio-economic disadvantage. 

 The topic is worthwhile to explore and the paper is nicely written, clearly outlining a robust approach. 

 I have a few comments that should be considered by the authors. 

 Firstly, I suspect that when articles are reviewed the title may prove a little problematic. Methodologically, many papers are likely to use area level disadvantage as a way to measure deprivation. If the review will only focus on *people* experiencing socio-economic disadvantage, then do you intend to exclude studies that only include area based measure of disadvantage, instead only selecting those that have some individual measure of socio-economic disadvantage? If so, that should be clearly stated in the protocol, if not, I suggest amending the title so it is not misleading. 

 I also wonder how utilitarian cycling will be determined. It does not appear in the keywords, and many studies will not distinguish between recreational and utilitarian cycling, so I think it would help to be clear about what will be included and excluded. At present, the article says that studies must explicitly focus on cycling for transport. I suspect this will be unclear in many articles, leading to exclusion of many studies. I also find the example of what would be excluded a little unlcear - physiological testing or warm-up exercise - what about a family leisure ride?  

 Consider changing keywords to depriv* as some studies may used deprived. Could also consider 'poverty', 'inclus*" 'equity' 'social exclusion'. 

 Finally, I found the introduction compelling and concise, with a nice framing of this as an environmental health issue. However, while the focus on mode shift is clear, given the focus population I think it would also be worthwhile acknowledging that supportive cycling environments can improve accessibility to destinations for lower income communities - who are already less likely to own a car. While the complex relationship between cycling and socioeconomic disadvantage is discussed, it is not clear that cycling may provide a mode of accessibility to non-car users, who currently face exclusions/missed journeys.

Is the study design appropriate for the research question?

Yes

Is the rationale for, and objectives of, the study clearly described?

Yes

Are sufficient details of the methods provided to allow replication by others?

Partly

Are the datasets clearly presented in a useable and accessible format?

Yes

Reviewer Expertise:

Transport and health, mixed methods, equity

I confirm that I have read this submission and believe that I have an appropriate level of expertise to confirm that it is of an acceptable scientific standard.
